# C4d Is an Independent Predictor of the Kidney Failure in Primary IgA Nephropathy

**DOI:** 10.3390/jcm13175338

**Published:** 2024-09-09

**Authors:** Nikola Zagorec, Ivica Horvatić, Dino Kasumović, Besa Osmani, Slavica Sović, Jagoda Nikić, Matija Horaček, Petar Šenjug, Krešimir Galešić, Danica Galešić Ljubanović

**Affiliations:** 1 Department of Nephrology and Dialysis, Dubrava University Hospital, 10000 Zagreb, Croatia; 2 Faculty of Pharmacy and Biochemistry, University of Zagreb, 10000 Zagreb, Croatia; 3 School of Medicine, University of Zagreb, 10000 Zagreb, Croatia; 4 Department of Medical Statistics, Epidemiology and Medical Informatics, School of Public Health “Andrija Štampar”, 10000 Zagreb, Croatia; 5 Nursing School Mlinarska, University of Applied Health Sciences, 10000 Zagreb, Croatia; 6 Department of Pathology, School of Medicine, University of Zagreb, 10000 Zagreb, Croatia; 7 Division of Nephropathology and Electron Microscopy, Department of Pathology and Cytology, Dubrava University Hospital, 10000 Zagreb, Croatia

**Keywords:** IgA nephropathy, kidney biopsy, immunohistochemistry, C4d, renal prognosis, complement system activation, lectin pathway, alternative pathway, renal survival, kidney failure

## Abstract

**Background:** C4d deposits are present in a substantial proportion of patients with IgA nephropathy (IgAN), indicating the activation of the lectin pathway (LP) of the complement system. It seems that patients with activated LP have worse renal prognosis. The aim of this study was to investigate the prevalence and prognostic significance of C4d in our cohort of patients with primary IgA nephropathy (pIgAN). **Methods:** Patients with pIgAN were recruited from a hospital register of kidney biopsies of the Department of Nephrology and Dialysis, Dubrava University Hospital, Zagreb. Additional immunohistochemistry staining for C4d was performed on paraffin-embedded kidney tissue, and patients were stratified into being C4d positive or C4d negative. The clinical and histologic features of patients were analyzed and compared regarding C4d positivity. The primary outcome was defined as kidney failure (KF), and predictor variables of KF and renal survival were analyzed. **Results:** Of a total of 95 patients with pIgAN included in the study, C4d was present in 43 (45.3%). C4d-positive patients had a higher value of systolic (*p* = 0.039) and diastolic (*p* = 0.006) blood pressure at diagnosis as well as higher 24 h proteinuria (*p* = 0.018), serum urate (*p* = 0.033), and lower eGFR (*p* < 0.001). C4d-positive patients had worse renal survival (*p* < 0.001), higher rates of disease progression to KF (*p* < 0.001), and higher proteinuria (*p* < 0.001) and lower eGFR (*p* < 0.001) at the last follow-up. Glomerular C4d was an independent predictor of disease progression to KF (HR = 5.87 [0.95 CI 1.06–32.44], *p* = 0.032). **Conclusions:** C4d is an independent predictor of disease progression in patients with pIgAN. C4d may be used as an additional marker of progressive disease course in IgAN. The therapeutic implications of C4d status in IgAN, particularly in terms of complement inhibitors application, are not yet known.

## 1. Introduction

IgA nephropathy (IgAN) is the most common primary glomerular disease worldwide [1]. Despite its nature of being a slowly progressive disease, a substantial proportion (20–40%) of patients will reach kidney failure (KF) during 10–20 years from diagnosis [2,3]. In countries with higher incidences of IgAN, like East Asian countries, IgAN is a major cause of KF in adults, significantly contributing to the burden of kidney diseases in general [2,4]. The clinical presentation of IgAN is heterogeneous, with erythrocyturia and subnephrotic-range proteinuria, which are usually asymptomatic, being the most common, along with arterial hypertension. Other presentations, like full-blown nephrotic syndrome or rapidly progressive glomerulonephritis, are less common [4]. Since the clinical course of IgAN is quite variable, from rather benign to disease progression to KF, it is important to try to recognize those patients with progressive disease. Baseline proteinuria seems to be the most important clinical variable associated with disease progression, but baseline estimated glomerular filtration rate (eGFR), arterial hypertension, and smoking status are also important [5]. Histologic variables at the time of kidney biopsy are related to disease outcome. The prognostic significance of histologic variables included in the Oxford classification of IgAN and its revision (MEST-C score) has been shown in many studies [6,7,8,9]. Additionally, interest in the prognostic significance of complement components in IgAN has increased over the last 15 years.

The complement system is apparently deeply involved in the pathogenesis of IgAN, and there are numerous pieces of evidence supporting this, i.e., histologic studies revealing complement components deposition within glomeruli (mesangium particularly) and blood and/or urine studies showing complement system activation [10]. The alternative pathway (AP) of the complement system is thought to be overactivated in the majority of patients with IgAN. IgAN is a typical immune complex-mediated disease, where the deposition of galactose-deficient IgA1 (Gd-IgA1), together with IgG antiglican antibodies (towards Gd-IgA1), within the mesangial space initiates glomerular inflammation, proliferation and, eventually, scarring. It has been shown in vitro that polymeric IgA1 can activate the AP of the complement system interacting with C3 [11]. Some genetic studies have shown that in some patients with IgAN and more severe clinical presentation, inherited susceptibility for AP dysregulation was present (e.g., variants in *C3* and *FH* genes) [12]. In patients with IgAN, elevated serum levels of complement factor H-related proteins (CFHRs) were found to be associated with local (glomerular) AP activation, given that CFHRs act as competitive antagonists of FH [13]. On the contrary, the deletion of genes coding for CFHRs had a protective role in the development of IgAN, strongly supporting the role of AP in pathogenesis of IgAN [13]. C3 is present in the majority of patients with IgAN, and although some studies showed its association with worse renal outcomes, its prognostic significance is not clear [10,12]. Decreased serum C3 as well as C3/C4 serum ratio in patients with IgAN was associated with progressive IgAN, suggesting C3 consumption and AP activation [14,15]. In a substantial proportion of patients with IgAN, the lectin pathway (LP) of the complement system is shown to be activated. In up to 50% of patients with IgAN, mannose-binding lectin (MBL) is codeposited with IgA1 in mesangium along with other components like MBL-associated serine proteinase 1 (MASP1) and MASP2 [10]. IgA1 deposited in mesangium may stimulate mesangial cell proliferation and the production of collectin-11, a crucial pattern-recognition molecule in LP activation [16]. It seems that once activated, LP may initiate or enhance AP activation through direct interaction between MASP2 and C3 [17]. Importantly, C1q deposits are either absent or present only in a minority of patients with IgAN (up to 20%), suggesting an insignificant role of the classical pathway (CP) of complement activation in IgAN. However, C4d mesangial deposits are present in 20–55% of patients with IgAN and are more common in those with progressive disease. In some studies that included large cohorts of patients with IgAN, C4d was a predictor of worse renal survival and disease progression to KF [3,10,18,19,20,21,22,23,24]. C4d is a split product of C4b during complement activation via LP and CP and a stable tissue marker of complement activation, given that it remains covalently bound to the cell surface where complement activation had taken place [25]. In IgAN, since C1q is mostly negative, C4d indicates the LP of complement activation. 

Exploring the role of the complement system in the pathogenesis of IgAN is crucial for the development of new therapeutic targets. IgAN is a disease where, except for steroids, there is no strong evidence supporting the efficacy of other immunosuppressive agents. Therefore, the need for new therapeutic agents is unmet, and soon, this gap could be bridged by complement system-interfering agents. Some of them are already being tested in clinical trials with promising results [17,26]. However, there are still many unknowns regarding the role of the complement system in the pathogenesis of IgAN and its prognostic implications. The aim of this study was to explore the prevalence of C4d in our cohort of patients with primary IgAN and evaluate its prognostic significance. We aimed to explore the patterns of complement system activation in patients with IgAN as well as the differences between patients regarding the pattern of complement activation.

## 2. Materials and Methods

### 2.1. Study Design and Patients

This is a monocentric cohort study. Patients with primary IgA (pIgAN) nephropathy were recruited from the registry of kidney biopsies (Department of Nephrology and Dialysis, Dubrava University Hospital, Zagreb) from January 2007 to December 2017 among those registered with a diagnosis of IgA nephropathy/vasculitis. Every patient was thoroughly evaluated for secondary causes of IgAN in order to strictly select those with pIgAN. The inclusion criteria were as follows: (a) at least 8 glomeruli available for light microscopy (in order to apply scoring according to Oxford classification) and (b) absence of known secondary causes of IgAN, like liver cirrhosis, inflammatory bowel disease, IgA vasculitis, infections caused by hepatitis viruses or HIV (human immunodeficiency virus) and the presence of other systemic autoimmune diseases [6,27]. Patients younger than 18 years of age and those with baseline eGFR < 20 mL/min/1.73 m^2^ or dialysis commencement at the time of kidney biopsy were not included. Patients whose biopsy samples had less than one non-globally sclerosed glomerulus available for immunohistochemistry (IHC) were additionally excluded from analysis (exclusion criteria). Relevant demographic, clinical, and laboratory patients’ data at the moment of diagnosis, as well as follow-up and treatment data, were obtained from medical records. At the moment of kidney biopsy, every patient gave written informed consent for kidney biopsy and participation in the research.

### 2.2. Methods and Definitions

For kidney function estimation, the CKD-EPI 2021 equation was employed, expressing eGFR in mL/min/1.73 m^2^. KF was defined as a permanent decline in eGFR < 15 mL/min/1.73 m^2^ or kidney replacement therapy (KRT), i.e., dialysis or kidney transplantation, commencement. The term KF was used instead of “end-stage kidney disease” or “end-stage renal disease” according to the recommended nomenclature by KDIGO [28]. Proteinuria was measured from collected 24 h urine. Nephrotic syndrome was defined as a triad of nephrotic range proteinuria (>3.5 g/day), hypoalbuminemia (serum albumin < 35 g/L), and the presence of peripheral edema. Erythrocyturia was defined as more than three red blood cells per high-powered field (RBC/HPF) found in urine sediment examination. Arterial hypertension was defined as the value of systolic blood pressure (SBP) ≥ 140 mmHg and/or diastolic blood pressure (DBP) ≥ 90 mmHg and/or antihypertensive agent in chronic therapy (indicated for arterial hypertension management) [29]. Treatment with angiotensin-converting enzyme inhibitors or angiotensin II receptor blockers was considered renin–angiotensin–aldosterone system inhibition (RAASi). Mean arterial pressure (MAP) was calculated according to the following formula: (SBP + 2xDBP)/3. Body mass index (BMI) was calculated as a ratio of body mass (kg) and the square of height in meters.

### 2.3. Histological Studies and C4d Immunohistochemistry

Every biopsy specimen was analyzed using light (LM), immunofluorescent (IF) and electron microscopy (EM) by an experienced renal pathologist at the moment of diagnosis. Relevant histological data were retrieved from previous pathological reports. Based on LM, a number of segmentally sclerosed glomeruli (SSG) and globally sclerosed glomeruli (GSG) was analyzed. The percentage of SSG and GSG was calculated as the ratio of SSG or GSG and the total number of non-globally sclerosed glomeruli available for LM. Interstitial fibrosis and tubular atrophy (IFTA) were expressed as the percentage of cortical tubulointerstitial area affected by interstitial fibrosis and tubular atrophy. On IF, positivity for IgM, IgG, IgA, C3 and C1q was analyzed and expressed semiquantitative as 0 (negative), 1+ (mild), 2+ (moderate), and 3+ (strong). Patients were considered positive for C3, IgM or IgG if the positivity on IF was graded as at least mild (1+).

For additional analysis of C4d deposits in biopsy specimens, IHC on paraffin-embedded tissue was employed. Two to three μm-thick sections were stained for C4d using a rabbit anti-human C4d primary antibody (Cell marque, USA, Rocklin). After being kept at 60 °C for 1 h in the thermostat, the sections were incubated with primary C4d antibody for 36 min at 37 °C. Rabbit amplifier (Amplifier, Roche Tissue Diagnostics, USA, Rocklin) was used as a secondary antibody. Staining was carried out in the Ventana BenchMark ULTRA system (Roche Tissue Diagnostics, USA) using the Ultraview Universal DAB Detection kit (Roche Tissue Diagnostics, USA). As a positive control for C4d, we used a kidney specimen of a transplanted patients’ with C4d-positive antibody-mediated rejection. Two experienced renal pathologists, blinded for patients’ baseline data and outcomes, independently analyzed IHC sections. A minimum of one non-GSG in every sample was required for C4d analysis, and patients with less than one non-GSG available for IHC were additionally excluded from analysis. According to previous relevant publications, clear C4d positivity within at least one glomerulus was required for the sample to be classified as C4d-positive [3,24,25]. Based on C4d positivity on IHC, patients were stratified into two groups: C4d-positive (C4d+) or C4d-negative (C4d−).

### 2.4. Follow-Up, Treatment, Renal Outcomes and Study Endpoints

After kidney biopsy, patients were treated and followed-up in our outpatient clinic. Treatment was guided according to good relevant practice, guidelines for the treatment of glomerular diseases and the personal judgment of the leading clinician [30,31]. All of the patients (except for those with intolerance for any reason) received RAASi after diagnosis. At every follow-up visit, eGFR and 24 h proteinuria were measured. Under the term immunosuppressive therapy (ISTH), the administration of steroids, cyclophosphamide (CPS), azathioprine, budesonide, or rituximab was included. A steroid course, applied during follow-up and at least 6 months after initial steroid treatment completion, was considered as repeated steroid treatment. The primary renal outcome was defined as KF. Renal survival time was considered from the date of kidney biopsy to the date of reaching KF or the last follow-up visit for those not reaching KF (same as follow-up time).

### 2.5. Statistical Analysis

Numerical variable distributions were assessed using the Kolmogorov–Smirnov test and expressed as mean ± standard deviation (SD) in the case of a normal distribution; otherwise, they were expressed as median with interquartile range (IQR). Groups were compared for means using Student’s *t*-test and medians using the Mann–Whitney U test. Qualitative variables are expressed as percentages, and group comparison was performed using χ^2^ or Fisher’s exact test, as appropriate. Correlations were analyzed using Spearman’s coefficient. Kaplan–Meier analysis was used for KF-free renal survival time estimation, with a log-rank test used for curve comparison. KF-free renal survival time was expressed as mean ± SD. To determine independent predictors of disease progression to KF, multiple Cox regression models were used with the inclusion of predictor variables with *p* < 0.1 in univariate Cox regression. Results of Cox regression are expressed as hazard ratios (HRs) with a 0.95 confidence interval (CI). To evaluate survival models, Harrell’s C is reported. Statistical software MedCalc v2022 was employed for statistical analysis, and *p* value < 0.05 was considered as significant.

## 3. Results

### 3.1. Clinical Features of Patients with Primary IgAN

In a defined period from January 2007 to December 2017, there were 255 patients with a leading diagnosis of IgAN or IgA vasculitis within a hospital registry of kidney biopsies at the Department of Nephrology and Dialysis, Dubrava University Hospital, Zagreb. After detailed analysis, 108 patients met the inclusion criteria. After C4d IHC analysis, an additional 13 patients were excluded due to an insufficient sample for IHC analysis (exclusion criteria). Table 1 shows relevant clinical and histologic features of the final 95 patients with primary IgAN. The median age at diagnosis (kidney biopsy) was 44.6 (IQR 32–52.2) years, and 68 (71.6%) patients were male. Seventy-one (74.7%) of the patients had arterial hypertension, sixty-one (64.2%) had RAASi in therapy, twenty-seven (27.3%) were smokers, and only three (3.2%) had diabetes mellitus type 2. Twenty-four (25.3%) of the patients had an episode of gross hematuria. Regarding kidney function, the median eGFR at diagnosis was 67 (IQR 49–84) mL/min/1.73 m^2^. According to KDIGO eGFR categories, 26 (27.3%) of the patients had CKD G1, 36 (37.9%) CKD G2, 15 (15.8%) G3a, 9 (9.5%) CKD G3b, and 9 (9.5%) were at stage G4. Median 24 h proteinuria was 2.0 (IQR 1–3.9) g/day, and nine (9.5%) of patients presented with full-blown nephrotic syndrome. Erythrocyturia was present in 89 (93.4%) of the patients, and 46 (48.4%) had more than 25 RBC/HPF.

### 3.2. Histologic Features of Patients with Primary IgAN

**Figure 1:**
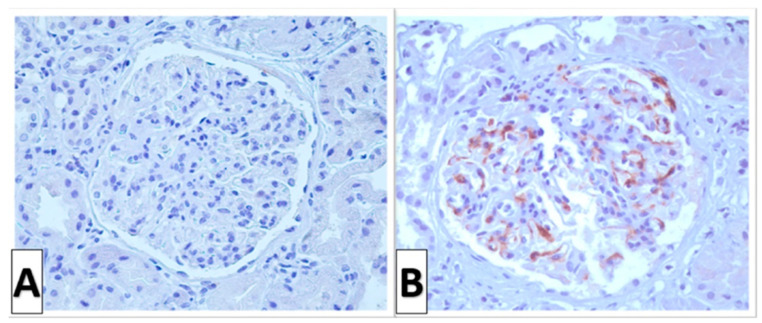
C4d immunohistochemistry in patients with IgA nephropathy (×400). (**A**) C4d-negative glomerulus in patients classified as being C4d negative. (**B**) C4d-positive glomerulus.

For LM analysis, a median of 20 (IQR 15–27) glomeruli were available. Of them, a median of 15% (IQR 6.9–27.2) were GSG, and 13.3% (6.4–25) were SSG. Table 2 shows relevant histologic features of patients with primary IgAN. According to Oxford classification, 54 patients (56.8%) had M1 score, 47 (49.5%) had E1 score, 75 (78.9%) had S1 score, 28 (29.5%) had T1 score, 10 (10.5%) had T2 score, and 48 patients (50.5%) had cellular or fibrocellular crescents, i.e., 39 (41%) had C1 score and 9 (9.5%) had C2 score. On IF, all patients were IgA positive, with the following semiquantitative intensity scores: 30 (31.6%), 1+; 37 (38.9%), 2+; and 28 (29.5%), 3+. Based on IHC analysis, 43 (45.3%) patients were classified as being C4d positive. Figure 1 shows the representative findings of IHC staining for C4d in our cohort.

### 3.3. Comparison of Baseline Features of Patients with Primary IgAN Regarding C4d Positivity

A comparison of baseline clinical and histologic features between C4d-positive and C4d-negative patients is shown in Table 1 and Table 2, respectively. Of clinical variables, C4d-positive patients had significantly higher values of SBP (135 [126–149] vs. 130 [120–140] mmHg, *p* = 0.039) and DBP (90 [80–99] vs. 80 [70–90] mmHg, *p* = 0.006). Kidney function (eGFR) was significantly lower in the C4d-positive group (53 [39.2–66.8] vs. 79 [61–95] mL/min/1.73 m^2^, *p* < 0.001), and 24 h proteinuria was significantly higher (2.7 [1.23–5.41] vs. 1.5 [0.86–2.95] g/day, *p* = 0.018). C4d-positive patients had higher serum uric acid levels (*p* = 0.033) and lower values of serum IgG (*p* = 0.034). Serum uric acid level negatively correlated with eGFR (ρ = −0.433, *p* < 0.001). C4d-positive patients had more pronounced chronic glomerular and tubulointerstitial changes: percentage of GSG (23.1 [12.5–41] vs. 10.2 [5.2–21.8]%, *p* < 0.001), percentage of SSG (18.8 [11–28.7] vs. 10 [4.4–20]%, *p* = 0.002), percentage of IFTA (30 [20–47.5] vs. 10 [7.5–22.5]%, *p* < 0.001), proportion of patients with score S1 (90.1 vs. 69.2%, *p* = 0.011), proportion of patients with score T1 (48.8 vs. 13.5%, *p* < 0.001) and score T2 (18.6 vs. 3.8%, *p* = 0.020). There was no significant difference in proportions of patients with scores M1, E1, C1 and C2 between C4d groups as well as for proportions of patients having C3, IgM and IgG deposits on IF.

### 3.4. Treatment Modalities

After kidney biopsy and diagnosis, all patients except one female (due to intolerance) were treated with RAASi. Initial steroid treatment was applied in 74 (77.9%) patients, and of them, 6 patients received a combination of steroids and CPS (Balardie–Robertson’s protocol) due to rapidly progressive clinical presentation or presence of crescents (Table 3). There was no difference in the proportion of patients treated with steroids between the C4d-positive and negative group (*p* = 0.216). However, patients with higher active Oxford scores received more commonly steroid treatment: E1 vs. E0 (89 vs. 66.7%, *p* = 0.008) and C2 (100%) and C1 (94.9%) vs. C0 (59.6%, *p* < 0.001). During follow-up, 14 (14.7%) patients received a second line of ISTH: CPS in ten (10.5%) patients, budesonide in four (4.2%), and rituximab in one patient with severe nephrotic syndrome previously treated with steroids and CPS. Taken together, 16 (16.8%) patients received CPS, 6 (6.5%) azathioprine, 4 (4.2%) budesonide and one (1%) rituximab, and, in total, 21 (21.1%) patients received additional ISTH on top of steroid treatment. Of 74 patients treated initially with steroids, 25 (33.4%) had repeated steroid courses. There was no significant difference in the steroid administration rate, additional ISTH administration rate, and the repeated steroid administration rate between C4d-positive and negative groups (Table 3).

### 3.5. Follow-Up, Disease Outcome and Renal Survival

**Figure 2:**
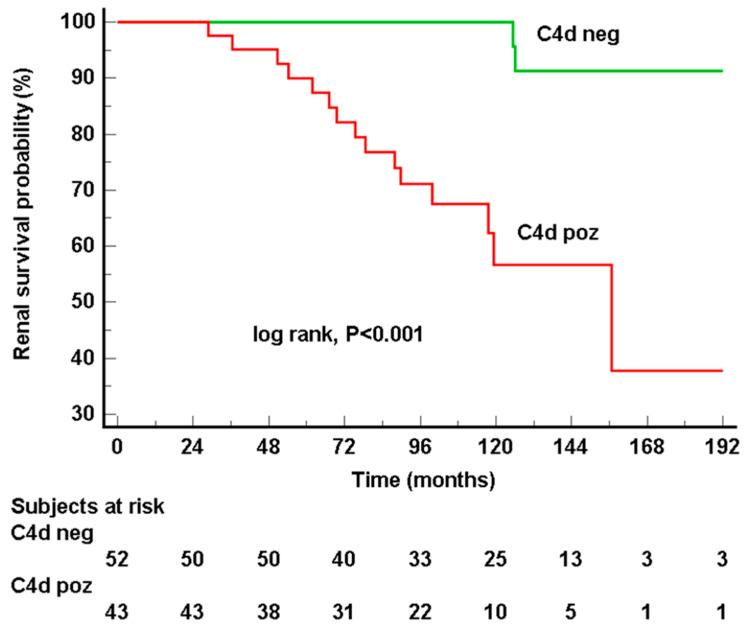
According to Kaplan–Meier analysis, C4d-positive patients had significantly worse KF-free renal survival time compared to C4d-negative patients (log-rank χ^2^ = 17 *p* < 0.001).

**Figure 3:**
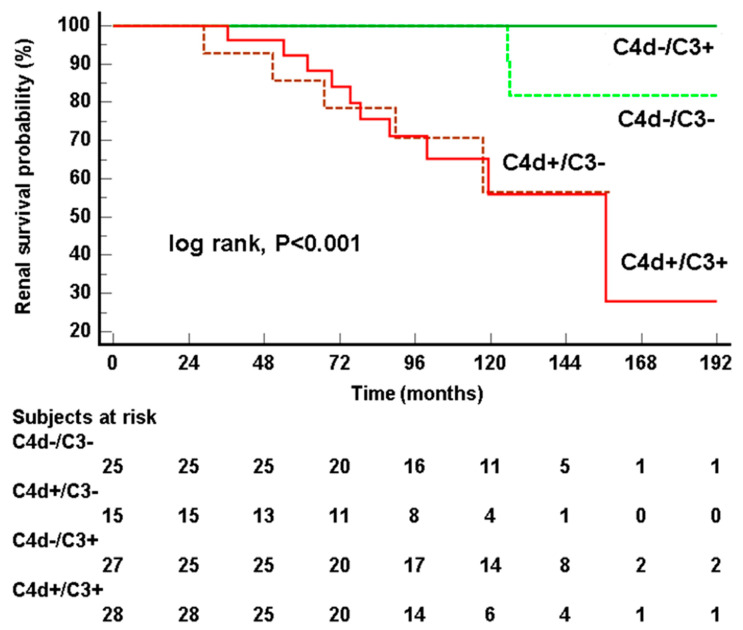
Patients with both C4d and C3 deposits (red lines) had similar kidney failure-free renal survival times as patients being only C4d-positive. C4d-negative patients (green lines) had significantly better kidney failure-free renal survival time than C4d-positive patients regardless of the presence of C3 (log-rank, χ^2^ = 17.6, *p* < 0.001). It seems that the presence of C3 deposits does not influence renal survival.

Median follow-up in our cohort was 102.2 (IQR 72.1–138.2) months. During the follow-up period, 17 (17.9%) of patients reached KF (two C4d-negative and 15 C4d-positive, *p* < 0.001). Of those who did not reach KF, C4d-positive patients had higher 24 h proteinuria (1 [0.51–1.8] vs. 0.35 [0.2–0.7] g/day, *p* < 0.001) and lower eGFR (without patients reaching KF, 52 [39–75] vs. 81 [68–102] mL/min/1.73 m^2^, *p* < 0.001) at the last follow-up (Table 3). According to Kaplan–Meier analysis, overall renal survival without KF was 174.9 ± 4.6 mo (143.3 ± 12.8 in the C4d-positive group vs. 197.9 ± 4.6 in the C4d-negative group, log-rank, χ^2^ = 17, *p* < 0.001) (Figure 2). Patients with a T2 score had the worst renal survival (102.8 ± 18.2 mo), followed by patients with a T1 score (160.2 ± 14.6 mo) and T0 score (192.2 ± 5.9 mo) (log-rank, χ^2^ = 21.5, *p* < 0.001, Figure S1). There were no significant differences in renal survival in other Oxford categories: M1 (138.5 ± 5.6 mo) vs. M0 (181.8 ± 8.9 mo, *p* = 0.361), E1 (135.1 ± 6.6 mo) vs. E0 (184.9 ± 7.9 mo, *p* = 0.119), S1 (170.2 ± 8.7 mo) vs. S0 (187.2 ± 11.3 mo, *p* = 0.316) and C2 (112.5 ± 81 mo) vs. C1 (145.5 ± 5.8 mo) and C0 (177.2 ± 8.9 mo, *p* = 0.232). Likewise, there was no significant difference in renal survival between the C3-positive (173.6 ± 9.6 mo) and negative (165.7 ± 9.3, *p* > 0.9) groups as well as between IgM-positive (137.8 ± 11.1 mo) and negative (178.9 ± 7.2, *p* = 0.353) groups. Interestingly, in a separate Kaplan–Meier analysis, combining C4d and C3 positivity, patients being both C4d and C3 positive had similar renal survival to those being only C4d positive (Figure 3). Similarly, the presence of C3 deposits did not influence renal survival in C4d-negative patients, and the difference in renal survival remained significant between C4d-positive and C4d-negative groups, regardless of C3 (log-rank, χ^2^ = 17.6, *p* < 0.001).

### 3.6. Identification of Predictors of Disease Progression in Primary IgAN

The Cox regression model was employed for independent predictors of disease progression identification (Table 4). In univariate analysis, the following variables were associated with disease progression to KF: SBP (HR per 1 mmHg increase 1.03 [0.95 CI 1.01–1.05], *p* = 0.008), DBP (HR per 1 mmHg increase 1.06 [0.95 CI 1.02–1.10], *p* = 0.006), 24 h baseline proteinuria (HR per 1 g/day increase 1.27 [0.95 CI 1.11–1.44], *p* < 0.001), serum uric acid level (HR per 1 μmol/L increase 1.008 [0.95 CI 1.003–1.01], *p* = 0.003), baseline eGFR (HR per 1 mL/min/1.73 m^2^ decrease 1.05 [0.95 CI 1.03–1.08], *p* < 0.001), T1 score (reference T0, HR 4.15 [0.95 CI 1.21–14.20], *p* = 0.023), T2 score (reference T0, HR 10.93 [0.95 CI 3.07–38.89], *p* < 0.001), and C4d positivity (reference C4d negative, HR 10.63 [0.95 CI 2.41–46.83], *p* = 0.002). Given that both SBP and DBP were associated with KF, MBP (HR per 1 mmHg increase 1.05 [0.95 CI 1.02–1.09], *p* = 0.003) was used in a multimodal Cox model. As an alternative to T score, the percentage of IFTA was analyzed separately (HR per 1% increase 1.05 [0.95 CI 1.02–1.07], *p* < 0.001). In the first multimodal Cox model (including MAP, IFTA, 24 h proteinuria, baseline eGFR, serum uric acid level and C4d status), independent predictors of disease progression to KF were as follows: 24 h proteinuria (HR per 1 g/day increase 1.31 [0.95 CI 1.10–1.58], *p* = 0.003), baseline eGFR (HR per 1 mL/min/1.73 m^2^ decrease 1.07 [0.95 CI 1.03–1.11], *p* = 0.005), serum uric acid level (HR per 1 μmol/L increase 1.006 [0.95 CI 1.00–1.01], *p* = 0.036), and C4d positivity (HR 5.71 [0.95 CI 1.08–30.19], *p* = 0.040) (Table 5). In the second model including T score instead of IFTA, independent predictors of disease progression remained the same: baseline eGFR (HR per 1 mL/min/1.73 m^2^ decrease 1.06 [0.95 CI 1.02–1.09], *p* = 0.011), 24 h proteinuria (HR per 1 g/day increase 1.29 [0.95 CI 1.10–1.52], *p* = 0.002), serum uric acid level (HR per 1 μmol/L increase 1.006 [0.95 CI 1.00–1.01], *p* = 0.027), and C4d positivity (HR 5.87 [0.95 CI 1.06–36.42], *p* = 0.042) (Table 5).

## 4. Discussion

Here, we showed that C4d, a tissue marker of complement activation in patients with pIgAN, is an independent predictor of worse renal survival and disease progression to KF. This is additional confirmation of some previously published results on the prognostic significance of C4d in IgAN [3,24].

The prevalence of C4d in our cohort of patients with pIgAN was 42.3%, which is similar to the results of previous studies that showed C4d positivity in 22–55% patients [3,18,24]. In those with glomerular C4d deposits, the LP of the complement system is activated, as drawn from previous studies. Apparently, LP is activated in less than 50% of patients with IgAN. C4d was also present in children with IgAN (prevalence was 36%) [32]. In the early stages of IgAN (patients with eGFR > 80 mL/min/1.73 m^2^), the prevalence of C4d was lower (20%), as shown in a study conducted by Segarra et al. [24]. It is not known at what point in IgAN development the complement system is activated and gets involved in pathogenesis, but this may be a dynamic process that is specific to each patient. It can be hypothesized that the higher prevalence of C4d in IgAN cohorts, including individuals with lower eGFR, reflects the later involvement of LP in disease pathogenesis. It was shown that all C4d-positive patients with IgAN remained positive on repeated kidney biopsies, and some C4d-negative became positive in the meantime [24]. Therefore, the prevalence of C4d positivity within a cohort of IgAN patients depends on patient features and the stage of disease at the time when a kidney biopsy is performed. Of note, the method employed for C4d analysis may influence the final results because IF seems to be a little more sensitive than IHC [3,33].

Interest in the role of the complement system in the pathogenesis of IgAN has been rising over the last 15 years. An initial study by Espinosa et al. showed that C4d is present in one-third of patients with IgAN and that they had worse renal survival [18]. A later study including a larger number of patients showed that C4d is an independent predictor of disease progression to KF [3]. Additionally, similar results were shown in patients with early-stage IgAN (eGFR > 80 mL/min/1.73 m^2^) and in children with IgAN or IgA vasculitis [19,24,32]. Except for KF, C4d was shown to be a predictor of eGFR slope as an outcome, sometimes a more sensitive measure of IgAN progression, which is a slowly progressive disease in itself [24]. In our cohort that included patients with eGFR > 20 mL/min/1.73 m^2^, C4d is confirmed to be an independent predictor of “hard outcomes” like KF in pIgAN. C4d-positive patients had more severe clinical presentation at the moment of diagnosis including higher arterial blood pressure, higher proteinuria, and lower eGFR, which is consistent with the results of previous studies [3,24,34]. Eventually, C4d-positive patients had lower eGFR and higher proteinuria at the last follow-up. Of note, in our cohort, C4d-positive patients had significantly higher serum uric acid levels. Given that uric acid levels correlated negatively with eGFR, higher levels of uric acid in C4d-positive patients could be due to lower eGFR. However, serum uric acid levels remained an independent predictor of KF in multimodal analysis, together with eGFR, 24 h proteinuria, and C4d positivity. Previously mentioned studies analyzing predictors of KF in IgAN did not include uric acid level in their analyses. Data on the prognostic significance of uric acid levels in IgAN are not consistent; however, some studies have shown its association with disease progression and more severe chronic histologic changes [15,35]. It was shown that urate crystals can activate the AP of the complement system and interfere with complement components [36]. Whether there is any association between uric acid and complement system activation in IgAN, it is difficult to say, and further investigations are needed to clarify this.

In our cohort, C4d-positive patients had more severe chronic histologic features, i.e., the percentage of GSG, SSG, and IFTA or, expressed through Oxford scores, a higher proportion of S1 score (vs. S0) and T1/T2 score (vs. T0). Patients with T2 and T1 scores had worse renal survival compared to those with T0 score; however, the T score was not a significant predictor of disease progression. A similar result was shown in a study conducted by Segarra et al., where C4d was included in the final analysis of the predictors of KF [24]. Oxford classification and the prognostic significance of defined scores within this scoring system have been validated in many studies, but C4d was not considered in this context [7,9]. The significance of C4d analysis in the context of Oxford classification is not clear, but C4d may be of additional value in IgAN prognostication. On the other hand, there was no significant difference in the active score (M, E and C) proportions between C4d-positive and negative patients, and neither were those scores associated with progression to KF. A possible explanation for such results may be the fact that patients with M1, E1 and C1/C2 scores received more common ISTH, with a greater probability of a positive effect of therapy since histologic lesions were active. Interestingly, there were no differences in the rate of steroid treatment and the rate of repeated steroid treatment between C4d-positive and negative groups in our cohort, similar to a report conducted by Espinosa et al. [3]. In a study conducted by Segarra et al. that included patients with early-stage IgAN, C4d-positive patients received more common treatments, such as prednisone and other ISTH [24]. While considering possible differences in the treatment modalities between C4d-positive and negative patients, great caution is needed, given that the treatments in all cohorts were not uniform.

Previous studies on complement system activation in IgAN are consistent regarding AP being activated in the vast majority of patients. Such conclusions rely on the presence of C3 (in up to 90% of patients) along with the other components of AP (factor B and H, CFHR1-3, etc.) in biopsy samples of patients with IgAN [10,12]. C3 is a central molecule of AP, and except for the probable direct activation of the AP by polymeric IgA1 deposited within mesangium, the activation of the AP may be enhanced via the interaction of LP components (MASP2 and MASP3) with C3 and factor D [11,17]. Reports on the prognostic significance of C3 in IgAN are not consistent [14]. Some studies have shown that lower serum C3 values and a lower C3/C4 ratio, as possible markers of C3 consumption, were associated with worse outcomes in IgAN [15,37]. In our study, lower serum C3 levels were associated with KF only in univariate Cox regression and the level of significance was borderline. Patients having C3 deposits on kidney biopsy (56.8% in our cohort) have similar outcomes as C3-negative patients. Interestingly, in a separate survival analysis, patients being both C4d and C3-positive had similar survival to those who were C4d-positive and C3-negative and were significantly worse than those who were C4d-negative, regardless of the presence of C3. That would indicate that the LP of complement activation (measured through C4d deposition) is a significant contributor to a more progressive IgAN course. C3 seems not to be relevant in IgAN prognostication, but some other components of AP activation, still not known, may be important in this context and remain to be investigated in the future. 

In our cohort, all patients were C1q-negative, which, in the constellation with C4d positivity, favors LP activation but not CP activation. Therefore, CP apparently does not play a significant role in the pathogenesis of IgAN. The prognostic role of C4d and LP of complement activation has been clearly shown in IgAN, but it is still not known why LP is activated only in one group of patients and why this is associated with worse disease outcomes. Regardless of that gap in our knowledge, it is important to identify those patients with a higher probability of disease progression because this may have implications for treatment and follow-up. In any case, the determination of C4d in IgAN is a widely available and relatively inexpensive method, which may become routine in the diagnostics of IgAN. It is not known if there are other serum or urine markers of LP activation that could replace tissue C4d analysis. Namely, it was shown that the urine C4d level is associated with the progression of IgAN, but the sensitivity and specificity of urine C4d regarding tissue C4d presence (discriminatory potential) is unknown [38]. Whether C4d-positive patients with IgAN should be treated differently in order to slow disease progression is not known. Ongoing clinical trials investigating the effect of factor B (iptacopan) and factor D inhibitors in IgAN show promising results [17]. Narsoplimab, a monoclonal antibody targeting MASP2 and the LP of complement activation, has already entered phase III of a clinical trial and indicated promising results [39]. We could hypothesize that patients with an activated LP of the complement system will respond better to LP inhibitors like narsoplimab. However, initial studies did not stratify patients regarding the presence of C4d deposits, and for this question to be answered, further investigation will be needed. Nonetheless, we are in an exciting era of complement system inhibitors that could fundamentally change the treatment of IgAN.

In conclusion, the presence of C4d in IgAN is associated with worse renal survival and disease progression to KF. Why the LP of the complement system is activated in only particular patients with IgAN remains to be clarified in further investigations as well as the therapeutic implications of such LP activation in IgAN.

## Figures and Tables

**Table 1:** Demographic and clinical features of patients with primary IgAN at the moment of diagnosis (kidney biopsy) and their comparison regarding C4d positivity.

Variable	Total (N = 95)	C4d− (N = 52)	C4d+ (N = 43)	*P*
Males (%)	68 (71.6)	37 (71.2)	31 (72.1)	>0.9 ^χ^
Age (years)	44.6 (32–52.2)	43.7 (31.9–54.3)	44.8 (33.9–51.5)	0.878 ^u^
Arterial hypertension (%)	70 (73.7)	35 (67.3)	35 (81.4)	0.123 ^χ^
Smoking (%)	27 (28.7)	14 (26.9)	13 (30.2)	0.669 ^χ^
RAASi at kidney biopsy (%)	60 (63.8)	35 (67.3)	25 (58.1)	0.437 ^χ^
SBP (mmHg)	130 (120–140)	130 (120–140)	135 (126–149)	**U = 845, 0.039** ^u^
DBP (mmHg)	85 (70–90)	80 (70–90)	90 (80–99)	**U = 761, 0.006** ^u^
BMI (kg/m^2^)	26.4 (23.7–29.8)	26.4 (23.6–31)	27.1 (23.9–29.1)	0.729 ^u^
Gross hematuria (%)	24 (25.3)	15 (28.8)	9 (20.9)	0.379 ^χ^
Hemoglobin (g/L)	136 (126–146)	136.5 (130–147)	136 (118–146)	0.607 ^u^
eGFR (mL/min/1.73 m^2^)	67 (49–84)	79 (61–95)	53 (39.2–66.8)	**U = 555, <0.001** ^u^
Serum urate (μmol/L)	413 (349–488)	389 (326–467)	446 (378–515)	**U = 741, 0.033** ^u^
Total cholesterol (mmol/L)	5.8 (4.88–7.24)	5.5 (4.78–7.33)	6.2 (5.2–7.17)	0.138 ^u^
LDL cholesterol (mmol/L)	3.55 (2.9–4.46)	3.46 (2.57–4.21)	3.8 (3–4.7)	0.355 ^u^
HDL cholesterol (mmol/L)	1.19 (1–1.5)	1.2 (1–1.6)	1.16 (1–1.4)	0.715 ^u^
Triglycerides (mmol/L)	1.9 (1.4–2.75)	1.66 (1.2–2.94)	2.27 (1.63–2.67)	0.081 ^u^
Serum albumin (g/L)	40 (37–43)	41 (37–44.5)	39 (36–42)	0.061 ^u^
Serum C3 (g/L)	1.22 (1.07–1.35)	1.22 (1.07–1.35)	1.22 (1.1–1.35)	>0.9 ^u^
Serum C4 (g/L)	0.33 (0.29–0.43)	0.33 (0.28–0.4)	0.37 (0.29–0.44)	0.145 ^u^
Serum IgA (g/L)	3.25 (2.39–4.33)	3.37 (2.38–4.53)	2.85 (2.48–3.89)	0.277 ^u^
Serum IgG (g/L)	10 (7.9–12.4)	11 (8.58–12.4)	8.97 (7.4–11.5)	**U = 693, 0.034** ^u^
Serum IgM (g/L)	0.91 (0.67–1.3)	0.86 (0.68–1.3)	0.92 (0.66–1.21)	0.767 ^u^
24 h proteinuria (g/day)	2 (1–3.87)	1.5 (0.86–2.95)	2.57 (1.23–5.41)	**U = 802, 0.018** ^u^

Abbreviations: N, number; *p*, *p* value; RAASi, renin–angiotensin–aldosterone system inhibitors; SBP, systolic blood pressure; DBP, diastolic blood pressure; BMI, body mass index; eGFR, estimated glomerular filtration rate; LDL, low-density lipoprotein; HDL, high-density lipoprotein; ^χ^ χ^2^ test; ^u^ Mann–Whitney U test.

**Table 2:** Histologic features of patients with primary IgAN and their comparison regarding C4d positivity.

Variable	Total (N = 95)	C4d− (N = 52)	C4d+ (N = 43)	*P*
N of glomeruli on LM	20 (15–27)	22.5 (15–27)	17 (13–24.8)	0.085 ^u^
GSG (%)	15 (6.9–27.2)	10.2 (5.2–21.8)	23.1 (12.5–41)	**U = 662, <0.001** ^u^
SSG (%)	13.3 (6.4–25)	10 (4.4–20)	18.8 (11–28.7)	**U = 700, 0.002** ^u^
IFTA (%)	20 (10–33.8)	10 (7.5–22.5)	30 (20–47.5)	**U = 547, <0.001** ^u^
M1 score (%)	54 (56.8)	25 (48.1)	29 (67.4)	0.059 ^χ^
E1 score (%)	47 (49.5)	23 (44.2)	24 (55.8)	0.264 ^χ^
S1 score (%)	75(78.9)	36 (69.2)	39 (90.1)	χ^2^ = 6.46, **0.011** ^χ^
T1 score (%)	28 (29.5)	7 (13.5)	21 (48.8)	χ^2^ = 14, **<0.001** ^χ^
T2 score (%)	10 (10.5)	2 (3.8)	8 (18.6)	χ^2^ = 5.38, **0.020** ^χ^
C1 score (%)	39 (41.1)	24 (46.2)	15 (34.9)	0.269 ^χ^
C2 score (%)	9 (9.5)	3 (5.8)	6 (13.9)	0.177 ^χ^
C3 deposits on IF (%)	54 (56.8)	27 (51.9)	27 (62.8)	0.289 ^χ^
IgM deposits on IF (%)	16 (16.8)	7 (13.5)	9 (2.9)	0.336 ^χ^
IgG deposits on IF (%)	6 (6.3)	2 (3.8)	4 (9.3)	0.405 ^f^

Abbreviations: N, number; *p*, *p* value; LM, light microscopy; GSG, globally sclerosed glomeruli; SSG, segmentally sclerosed glomeruli; IFTA, interstitial fibrosis and tubular atrophy; IF, immunofluorescence; ^χ^ χ^2^ test; ^u^ Mann–Whitney U test; ^f^ Fisher’s exact test.

**Table 3:** Treatment and outcomes of patients with primary IgAN and their comparison regarding C4d positivity.

Treatment and Outcomes	Total (N = 95)	C4d− (N = 52)	C4d+ (N = 43)	*P*
RAASi (%)	94 (98.9)	51 (98.1)	43 (100)	>0.9 ^χ^
Steroids (%)	74 (77.9)	38 (73.1)	36 (83.7)	0.216 ^χ^
CPS (total, %)	16 (16.8)	7 (13.5)	9 (20.9)	0.333 ^χ^
Steroids + CPS (%)	6 (6.3)	4 (7.7)	2 (4.7)	0.544 ^χ^
Other ISTH (%)	20 (21.1)	8 (15.4)	12 (27.9)	0.103 ^χ^
Repeated steroid administration (N = 74, %)	25/74 (33.8)	11/38 (28.9)	14/36 (38.9)	0.366 ^χ^
Median follow up (mo)	102.2 (72.1–138.2)	111.4 (74.4–144.8)	96.3 (7.7–118.9)	0.096 ^u^
Renal survival (mo)	174.9 ± 4.6	197.9 ± 4.6	143.3 ± 12.8	χ^2^ = 17, **<0.001** *
Progression to KF (%) ^f^	17 (17.9)	2 (3.8)	15 (34.9)	**<0.001** ^f^
eGFR at last follow-up (N = 78, mL/min/1.73 m^2^)	74 (43–94)	81 (68–102) (N = 50)	52 (39–75) (N = 28)	**U = 456, <0.001** **
24 h proteinuria at last follow-up (g/day)	0.58 (0.3–1.22)	0.35 (0.2–0.7)	1 (0.51–1.8)	**U = 396, <0.001** ^χ^

Abbreviations: N, number; *p*, *p* value; RAASi, renin–angiotensin–aldosterone system inhibitors; CPS, cyclophosphamide; ISTH, immunosuppressive therapy; KF, kidney failure; ^χ^ χ^2^ test; ^u^ Mann–Whitney U test; ^f^ Fisher’s exact test; * Kaplan–Meier analysis (mean ± SD); ** excluding those patients who reached KF.

**Table 4:** Identification of clinical and histologic variables associated with progression of primary IgAN to kidney failure (univariate Cox regression).

Clinical Variable	Univar. Cox Regression		Histologic Variable	Univar. Cox Regression	
	HR (0.95 CI)	*P*		HR (0.95 CI)	*P*
**Female sex**	1.38 (0.51–3.75)	0.526	IgM+ (ref. IgM-)	1.69 (0.55–5.19)	0.362
Age (per 1 year increase)	1.01 (0.97–1.05)	0.593	IgG+ (ref. IgG-)	2.13 (0.28–16.34)	0.468
SBP (per 1 mmHg increase)	1.03 (1.01–1.05)	**0.008**	C3+ (ref. C3-)	1.10 (0.42–2.89)	0.846
DBP (per 1 mmHg increase)	1.06 (1.02–1.10)	**0.006**	M1 score (ref. M0)	1.68 (0.62–4.55)	0.305
MAP (per 1 mmHg increase)	1.05 (1.02–1.09)	**0.003**	E1 score (ref. E0)	2.19 (0.81–5.96)	0.341
Arterial hypertension at diagnosis (yes)	2.21 (0.50–9.67)	0.294	S1 score (ref. S0)	2.05 (0.47–8.89)	0.341
RAASi at diagnosis	0.46 (0.18–1.19)	0.109	T1 score (ref. T0)	4.15 (1.21–14.20)	**0.023**
Smoker (yes)	1.50 (0.55–4.08)	0.429	T2 score (ref. T0)	10.93 (3.07–38.89)	**<0.001**
Gross hematuria (yes)	1.56 (0.57–4.26)	0.382	IFTA (per 1% increase)	1.05 (1.02–1.07)	**<0.001**
24 h proteinuria (per 1 g/day increase)	1.27 (1.11–1.44)	**<0.001**	C1 score (ref. C0)	0.86 (0.29–2.58)	0.788
eGFR (per 1 mL/min/1.73 m^2^ decrease)	1.05 (1.03–1.08)	**<0.001**	C2 score (ref. C0)	2.49 (0.65–9.45)	0.182
Serum C3 (per 1 g/L decrease)	10.75 (0.55–200)	0.116	C4d+ (ref. C4d−)	11.89 (2.7–52.4)	**0.001**
SerumC4 (per 1 g/L increase)	44.9 (0.37–5413)	0.119			
Serum IgA (per 1 g/L increase)	1.10 (0.80–1.51)	0.548			
Serumski IgG (per 1 g/L increase)	0.85 (0.71–1.005)	0.057			
Serum urate (per 1 μmol/L increase)	1.008 (1.003–1.01)	**0.003**			

Abbreviations: HR, hazard ratio; CI, confidence interval; *p*, *p* value; SBP, systolic blood pressure; DBP, diastolic blood pressure; MAP, mean arterial pressure; RAASi, renin–angiotensin–aldosterone system inhibitors; IFTA, interstitial fibrosis and tubular atrophy.

**Table 5:** Identification of independent predictor variables of primary IgAN progression to kidney failure using two different models—either with IFTA (model 1) or T score (model 2) (multimodal Cox regression).

Model 1			Model 2		
Variable	HR (0.95 CI)	*P*	Variable	HR (0.95 CI)	*P*
MBP (per 1 mmHg increase)	1.04 (0.9–1.09)	0.076	MBP (per 1 mmHg increase)	1.03 (0.99–1.09)	0.136
eGFR (per 1 mL/min/1.73 m^2^ decrease)	1.07 (1.03–1.11)	**0.005**	eGFR (per 1 mL/min/1.73 m^2^ decrease)	1.06 (1.02–1.09)	**0.011**
24 h proteinuria (per 1 g/day increase)	1.32 (1.09–1.58)	**0.001**	24 h proteinuria (per 1 g/day increase)	1.29 (1.10–1.52)	**0.002**
Serum urate (per 1 μmol/L increase)	1.006 (1.00–1.01)	**0.036**	Serum urate (per 1 μmol/L increase)	1.006 (1.00–1.01)	**0.027**
IFTA (per 1% increase)	0.99 (0.96–1.02)	0.570	T2 score (ref. T0)	1.41 (0.43–4.59)	0.569
C4d+ (ref. C4d−)	5.71 (1.08–30.19)	**0.040**	C4d+ (ref. C4d−)	5.87 (1.06–32.42)	**0.031**
Harrell’s C index: 0.917 (0.95 CI 0.87–0.96)			Harrell’s C index: 0.919 (0.95 CI 0.87–0.97)		

Abbreviations: HR, hazard ratio; CI, confidence interval; *p*, *p* value MAP, mean arterial pressure; IFTA, interstitial fibrosis and tubular atrophy.

## Data Availability

The data presented in this study are available upon request from the corresponding author due to privacy and ethical reasons.

## Supplementary Materials

The following supporting information can be downloaded at: https://www.mdpi.com/article/10.3390/jcm13175338/s1, Figure S1: According to Kaplan–Meier analysis, patients with T2 score had significantly worse kidney failure-free renal survival time than patients with T1 score and T0 score (log-rank, *p* < 0.001).

## Study Limitations

This study was conducted in only one center and the data were collected retrospectively. The treatment modalities used in our patients were not uniform across the whole cohort, and although they were guided by relevant treatment guidelines (KDIGO Glomerular Diseases) and good clinical practice, treatment choice may have been influenced by the individual preference of the responsible clinician [30]. Given that complement activation in IgAN is a dynamic process, there is a possibility that some C4d-negative patients may become C4d-positive at a later stage of the disease, as shown in a study conducted by Segarra et al. However, in separate analyses, the reclassification of some C4d-negative patients into C4d-positive did not have any impact on the final study results [24].
