# Supplementary material for: C4d Is an Independent Predictor of the Kidney Failure in Primary IgA Nephropathy
Source: J Clin Med. 2024 Sep 9;13(17):5338. doi: 10.3390/jcm13175338 (PMC11395978; doi:10.3390/jcm13175338)
Supplement: Supplementary file 1 [file jcm-13-05338-s001.zip › jcm-3166346-supplementary.pdf]

Supplementary Material:

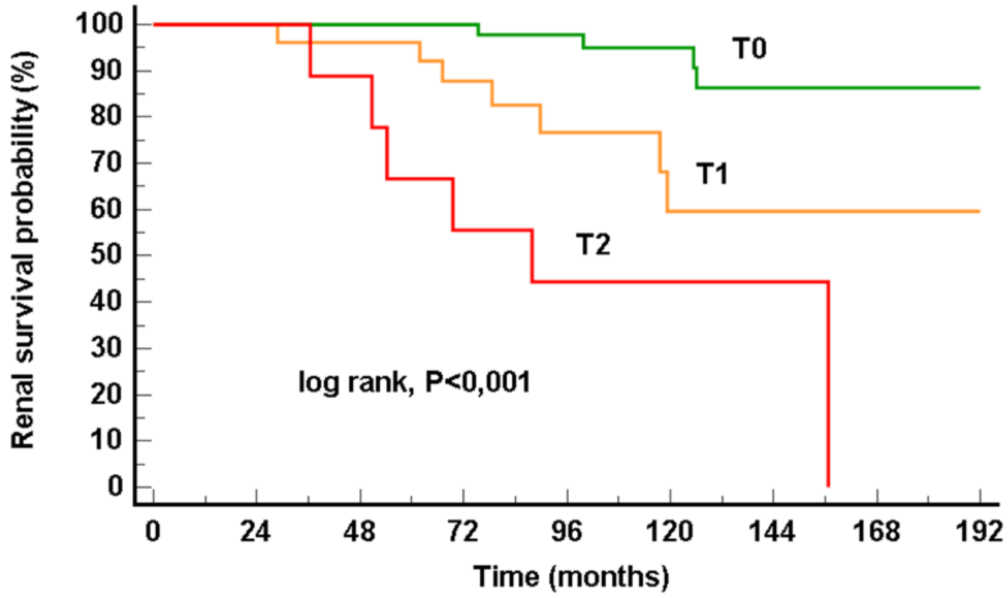

Subjects at risk

T0	57	56	56	47	39	25	11	3	3
T1	28	28	24	19	12	7	6	1	1
T2	10	9	8	5	4	3	1	0	0

Figure S1. According to Kaplan-Meier analysis, patients with T2 score had significantly worse kidney failure-free renal survival time than patients with T1 score and T0 score (log rank, χ^2=21.5, P<0.001).
